# ‘Fast cast’ and ‘needle Tenotomy’ protocols with the Ponseti method to improve clubfoot management in Bangladesh

**DOI:** 10.1186/s13047-017-0231-4

**Published:** 2017-11-09

**Authors:** Angela Evans, Mamun Chowdhury, Sohel Rana, Shariar Rahman, Abu Hena Mahboob

**Affiliations:** 1 Discipline of Podiatry, College of Science, Health, and Engineering, La Trobe University, Bundoora, Melbourne, Australia; 2 Road No 15, House 4, Block D, Banani, Dhaka, 1213 Bangladesh; 3 Mymensingh Medical College Hospital, Mymensingh, Bangladesh; 4 Dhaka Medical College Hospital, Dhaka, Bangladesh

**Keywords:** Clubfoot, Evidence, Ponseti, Tenotomy, Bangladesh

## Abstract

**Background:**

The management of congenital talipes equino varus (*clubfoot deformity*) has been transformed in the last 20 years as surgical correction has been replaced by the non-surgical Ponseti method. The Ponseti method, consists of corrective serial casting followed by maintenance bracing, and has been repeatedly demonstrated to give best results - regarded as the ‘gold standard’ treatment for paediatric clubfoot.

**Methods:**

To develop the study protocol Level 2 evidence was used to modify the corrective casting phase of the Ponseti method in children aged up to 12 months. Using Level 4 evidence, the percutaneous Achilles tenotomy (PAT) was performed using a 19-gauge needle instead of a scalpel blade, a technique found to reduce bleeding and scarring.

**Results:**

A total of 123 children participated in this study; 88 male, 35 female. Both feet were affected in 67 cases, left only in 22 cases, right only in 34 cases. Typical clubfeet were found in 112/123 cases, six atypical, five syndromic. The average age at first cast was 51 days (13–240 days).

The average number of casts applied was five (2–10 casts). The average number of days between the first cast and brace was 37.8 days (10–122 days), including 21 days in a post-PAT cast. Hence, average time of corrective casts was 17 days.

Parents preferred the reduced casting time, and were less concerned about unseen skin wounds.

PAT was performed in 103/123 cases, using the needle technique. All post tenotomy casts were in situ for three weeks. Minor complications occurred in seven cases - four cases had skin lesions, three cases disrupted casting phase. At another site, 452 PAT were performed using the needle technique.

**Conclusions:**

The ‘fast cast’ protocol Ponseti casting was successfully used in infants aged less than 8 months. Extended manual manipulation of two minutes was the essential modification. Parents preferred the faster treatment phase, and ability to closer observe the foot and skin. The treating physiotherapists preferred the ‘fast cast’ protocol, achieving better correction with less complication. The needle technique for PAT is a further improvement for the Ponseti method.

## Background

The management of congenital talipes equino varus (*clubfoot deformity*) has been transformed in the last two decades as surgical correction has been replaced by the non-surgical Ponseti method [1–3]. The Ponseti method, consists of corrective serial casting, a percutaneous Achilles tenotomy, followed by maintenance bracing [4]. This method has been repeatedly demonstrated to give the best results and is globally regarded as the ‘gold standard’ treatment for paediatric clubfoot [5–9].

Approximately 80% of children with congenital clubfoot deformity reside in low and middle income countries (LMIC’s - http://globalclubfoot.com/). Untreated the child with a clubfoot becomes increasingly disabled, suffers social stigma, pain, and poverty. The low-cost Ponseti method is applicable to clubfoot deformity globally, and particularly relevant in LMIC’s where disability will usually diminish individual productivity.

### The original Ponseti method

The original method described by Ponseti involves a series of plaster casts changed weekly for a period of six weeks, followed by percutaneous elongation of the Achilles tendon (PAT), and application of a further ‘healing’ cast for three weeks. The foot abduction bracing phase, is commenced immediately after the tenotomy cast is removed [10].

### Evidence for more frequent cast changes

Level 2 evidence indicates that accelerated cast changes has comparable outcomes to the original Ponseti method [11] with the benefit of less plaster cast immobilisation during the corrective phase.

A decade ago a non-randomised study found that casts could be changed every five days, rather than every seven days, with the same results, and saving ten to 12 days of plaster cast immobilisation [12]. Since then two RCT’s concluded that casts changed twice/week (even three times/week) give the same results as weekly casts. [11, 13]. Halving the casting phase from six to three weeks, has clear advantages. Less time immobilised in plaster casts is preferable for the child, and parents. Shorter time per cast reduces the likelihood of skin wounds.

It has been repeatedly shown that positive correlation exists between good use of the brace and less relapse of clubfoot correction. Starting the boots and bar habit earlier in infancy may be helpful [14, 15]. Practically, shortening the corrective phase lessens time away from work and home for parents, and means that awkward bathing and carrying has a shorter duration.

### Evidence for modifying the percutaneous Achilles tenotomy technique

Level 4 evidence indicates that the use of a needle rather than a scalpel blade, may be advantageous for severing the Achilles tendon percutaneously. A number of reports advocating the use of PAT using the needle technique have now appeared in the literature, and are positive in recommendation [16–18]. The advantages are ease of technique, reduced complications (bleeding), minimal scarring, reduced cost as an outpatient procedure using local anaesthesia.

‘Walk for Life’ (WFL) is a large-scale clubfoot project in Bangladesh that has provided treatment for 19,500 children since 2009 (www.walkforlifeclubfoot.org). WFL has been diligent in evaluating its outcomes and sharing these findings through publication, so that other clubfoot projects may be informed of findings [8, 19].

Good communication between WFL physiotherapists, supporting Bangladeshi medical staff, and WFL international volunteer advisors culminated in the development of the new protocol for Ponseti casting by one author (AE), and included the modified tenotomy technique developed by author SRah [18].

## Methods

Using Level 2 evidence, and previous field work [20], we developed a modified protocol for the casting phase of the Ponseti method in children aged up to 12 months (Table 1).

**Table 1:** Level 2 evidence and the development of the ‘fast cast’ protocol for typical clubfeet

Author, date	No. Participants = n	Avg age at start	Cast changed	Avg days in casts - ‘fast’ group	Tenotomy rate	Follow up	Comment
Morecuende, 2005	230 (non-random)	Not stated	5 days	30	85%	Study ran over 11 years	Older study, NR sample
Harnett, 2011	40 (RCT)	21 days	3 / week	16	79%	Min 6 mths Ave 8 mths	Youngest children
Xu, 2011	26 (RCT)	92 days	2 / week	20.6	87%	2–6 years	*Manipulation time of 2 min*
Ullah, 2014	28 (RCT)	18 weeks	2 / week	23.3	80%	Not reported	
Elgohary, 2015	41 (RCT)	11 weeks (SD 7)	2 / week	18.1 (SD 3)	91%	12–48 mths	Relapse rate 15% both groups
RCT data summary	RCTs *n* = 135	11 weeks	2/week	19.5 days	84% average	1 year (3 RCTs)	Young infants, 2 min. Stretch

Corrective casts were changed twice/week in babies aged 0 to 12 weeks of age, using a longer manipulation/stretch time of two minutes.NB ‘fast casts’ applies only to treatment casts; post-tenotomy cast is required for 3 weeks

Modifying the Ponseti protocol, corrective casts were changed every three days, with a single post-tenotomy cast for the standard 3 weeks.

A longer manual manipulation time of two minutes, was used prior to the application of each cast.

In addition, the PAT was performed using a 19-gauge needle, rather than a scalpel blade, an adapted technique found to reduce bleeding [17, 18]. All tenotomies were performed with minimal infiltration of local anaesthesia.

The study was carried out at the Mymensingh Medical College Hospital ‘Walk for Life’ clinic from June 2015 to June 2016.

In addition, PAT data using the needle method, was collected at Dhaka Medical College Hospital ‘Walk for Life’ clinic between 7 May 2013 and 1 August 2015, by one author (SRah).

Alongside the main inquiry of this study, we also assessed affluence using proxy indicators eg housing materials, Father’s monthly earnings – to gain insight regarding the practical factors which can influence access to children’s health care.

## Results

In total, 123 children participated in this study: 88 male, 35 female. Both feet were affected in 67 cases, left only in 22 cases, right only in 34 cases. In 112/123 cases there was typical clubfoot, six atypical or complex, five syndromic. The average age of children at first cast application was 51 days (13–240 days). Initial clubfoot severity scores were comparable across the typical (left 5/6; right 6/6), atypical (left 5.5/6; right 6/6), and syndromic (left 5/6; right 5/5) clubfeet.

**Fig. 1:**
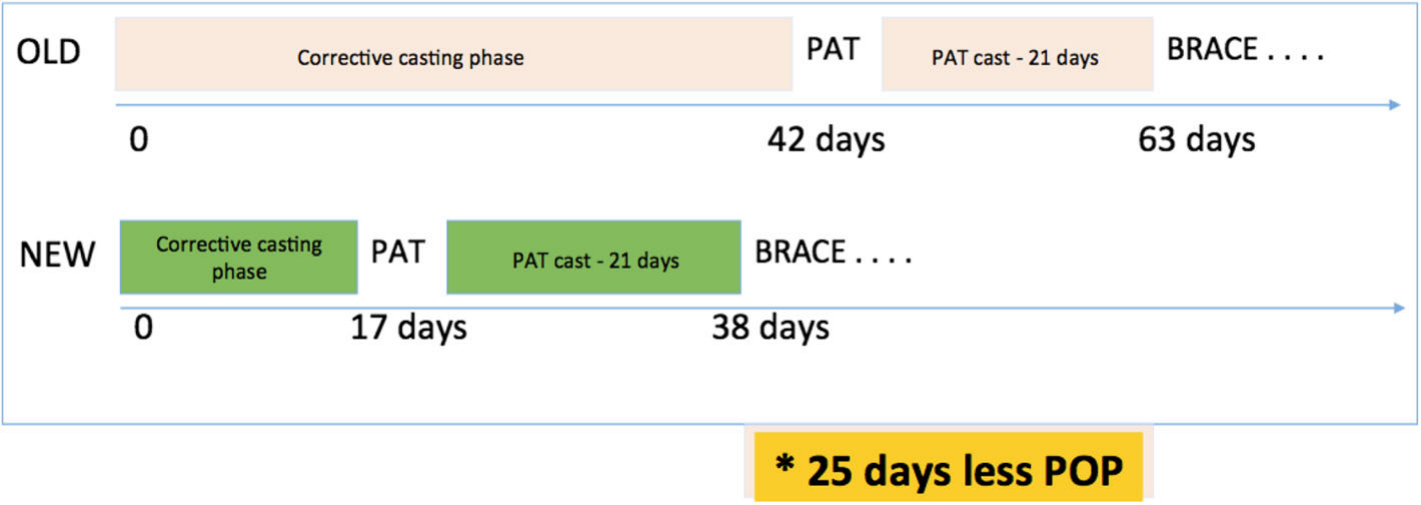
Comparison timelines of the traditional Ponseti method with the new ‘fast casts’ method. On average, children had 25 days less plaster cast immobilization with ‘fast casts’

The average number of casts applied was five (2–10 casts). The average number of days between the first cast and brace was 37.8 days (10–122 days), with 21 days in a post-tenotomy cast. Hence, average time of corrective casts was 17 days (Fig. 1). Minor complications with casting occurred in seven cases: four cases had skin lesions, three cases had a disrupted casting phase.

Clubfoot types showed some variation in treatment course: typical clubfeet (*n* = 112) average number of corrective casts(SD) 5(1.4), range 2–9 casts; no complications occurred; atypical clubfeet (*n* = 6) average number of corrective casts(SD) 6(3.5), range 4–12 casts; five complications occurred; syndromic clubfeet (*n* = 5) average number of corrective casts(SD) 5(1.8), range 3–8 casts; four complications occurred.

Achilles tenotomy was performed in 103/123 cases, using the needle technique. All post tenotomy casts were in situ for three weeks.

Between May 2013 and August 2015, 452 PAT using the needle technique, were performed at Dhaka Medical College Hospital (SRah). Of the total 452 cases, 238 were bilateral clubfoot cases, 214 were unilateral clubfoot. The babies were predominantly male (*n* = 276), with less females (*n* = 176). Minor complications in 11 cases (mostly bleeding due to aberrant blood vessel) and all were managed locally with pressure applied until bleeding ceased.

The proxy indicators of affluence were collated as follows: housing materials were either tin 109/123, or brick 14/123; Father’s earnings of 10,000 Taka or less/month (US$120) 76/123, 20,000 Taka (US$240) or more/month 47/123. In 95/123 families, there was only one income source.

Parents preferred the reduced casting time, and were less concerned about unseen skin wounds.

Physiotherapists also preferred the more frequent cast changes, observing less cast softening, and hence a better maintained corrective position of the foot.

## Discussion

The Ponseti method is the ‘gold standard’ technique for congenital clubfoot deformity. The ‘Fast Cast’ protocol is an evidence-based modification, used successfully by WFL in Bangladesh, and is preferred by both parents and treating clinicians, which is an important facet of shared decision making.

WFL has now adopted the ‘fast cast’ protocol wherever possible in children under 12 months of age.

Clinic logistics are a relevant factor in the use of the ‘fast cast’ protocol, and may present a challenge for entrenched weekly clubfoot clinics. This is a current reality for WFL, where each clinical team (physiotherapist and assistant) operate across a hub-and-spoke style clinic locations, with 4/7 days at a main clinic and one day per week at two smaller satellite clinics. Currently, the ‘fast cast’ protocol is only feasible at the larger clinics. The reason for the satellite clinics is to reduce travel distances for parents, most of whom are poor, and travel imposes a significant cost.

The basis of Ponseti method correction is manual – ‘fast casts’ relies on the usual accuracy of palpation and positioning, plus an extended *hold and stretch* time of two minutes, to elongate the ligaments and gain maximum correction/cast.

Clinicians are advised to maximize manual manipulation and positional stretch to gain the most available correction per cast. Rushing the pre-cast *hold and stretch* is likely to necessitate more casts, which increases the child’s immobilization time, and uses more resources.

More frequent cast changing means that each cast holds the manually gained corrected position firmly, with less time to soften, which is especially relevant in humid environs.

The parents’ preference for more frequent cast changes was expressed to treating clinicians, and specifically, parents reportedly liked visualising and washing their child’s foot and leg at shortened intervals. Whilst the number of casts and associated clinic visits for the corrective casting still averaged five, the shortened treatment phase may reduce the likelihood of treatment being interrupted, which has previously been reported as a factor reducing parental satisfaction [21].

Needle PAT advantages are supported by a number of clinical case series [18, 22, 23], and more recently, by a prospective randomized clinical trial [16]. The RCT (*n* = 55, average age at time of tenotomy 9.5 weeks) concluded no differences between the outcome of the blade versus needle groups at 1 year follow up [16]. Lessened complications and reduced parental anxiety are clear advantages of the needle technique. WFL are organising training workshops for doctors to increase the use of this method across Bangladesh.

Rahman et al. [18] presented a case series (*n* = 52) of PAT using needle method in children aged between 1 and 30 months, and reported a complication rate of 9/70 procedures, with bleeding, uncorrected foot position, and difficult procedure beingcited.

As indicated by the proxy indicators of affluence in this study, many families in Bangladesh are too poor to pay for their child’s clubfoot treatment. Without treatment, clubfoot deformity assures lifetime disability. WFL donors transform the lives of affected children. Since 2009, WFL has treated over 19,500 children with clubfoot deformity, and have demonstrated very good results [21].

### The possibility of further modifications to Ponseti’s original method

Whilst the Ponseti method is a demonstrably effective, and economical, it is still largely used with ‘Shotgun’ approach, rather than specifically targeting the presentation of individual cases. This is especially true of the challenging brace maintenance, which is advisably continued until a child is at least four years of age.

The identification of clubfoot sub-types, may in the future, allow for more individualized use of the Ponseti method. Whilst initial severity scores have been shown to provide predictability for the number of casts, and the need for a tenotomy, the period required for bracing to prevent deformity relapse remains a conundrum. Questions pending include: would active dorsiflexion mobilizing exercises during the brace phase assist, invoking French method principles; would encouraging a squat posture for play, or even a standing/sitting wedge, reduce the length of brace time required? These observations arise from our Bangladeshi children who routinely squat to play, eat and toilet – and where the relapse rate appears low, despite the usual tapering of brace use with time; how many months is the maintenance brace required (versus advised), and how is this determined?

## Conclusions

The ‘fast cast’ protocol Ponseti casting was successfully used in infants aged less than 8 months at treatment commencement. An extended two minute manual manipulation was the essential modification to the original method.

The parents preferred the faster treatment phase, and ability to observe their child’s foot and skin. The physiotherapists also preferred the ‘fast cast’ protocol, as casts were less likely to soften or break, providing better correction, and there were less adverse outcomes viz. skin sores, infections, joint stiffness, muscle wasting.

The needle technique for PAT has also been successfully implemented.

The main benefits are: less complications and scarring following the PAT and reduced plaster immobilisation – on average, 25 days less than with the traditional method; more effective casts, less skin wounds; and happier parents.

## Abbreviations

LMIC: Low and Middle Income Countries
MMCH: Mymensingh Medical College Hospital
PAT: Percutaneous Achilles Tenotomy
RCT: Randomised Controlled Trial
WFL: Walk for Life
